# Enhanced production of hyoscyamine and scopolamine from genetically transformed root culture of *Hyoscyamus reticulatus* L. elicited by iron oxide nanoparticles

**DOI:** 10.1007/s11627-017-9802-0

**Published:** 2017-02-27

**Authors:** Fereshte Moharrami, Bahman Hosseini, Ali Sharafi, Manouchehr Farjaminezhad

**Affiliations:** 1 Horticultural Sciences Department, Faculty of Agriculture, Urmia University, Urmia, Iran; 2 Pharmaceutical Biotechnology Research Center, School of Pharmacy, Zanjan University of Medical Sciences, Zanjan, Iran; 3 Medicinal Plants Research Center, Ardabil Branch, Islamic Azad University, Ardabil, Iran

**Keywords:** Elicitation, Hairy root, *Hyoscyamus reticulatus* L., Iron oxide nanoparticles, Tropane alkaloids

## Abstract

The medicinal plant *Hyoscyamus reticulatus* L. is a rich source of hyoscyamine and scopolamine, the tropane alkaloids. The use of hairy root cultures has focused significant attention on production of important metabolites such as stable tropane alkaloid production. Elicitation is an effective approach to induce secondary metabolite biosynthetic pathways. Hairy roots were derived from cotyledon explants inoculated with *Agrobacterium rhizogenes* and elicited by iron oxide nanoparticles (FeNPs) at different concentrations (0, 450, 900, 1800, and 3600 mg L^−1^) for different exposure times (24, 48, and 72 h). The highest hairy root fresh and dry weights were found in the medium supplemented with 900 mg L^−1^ FeNPs. Antioxidant enzyme activity was significantly increased in induced hairy roots compared to non-transgenic roots. The highest hyoscyamine and scopolamine production (about fivefold increase over the control) was achieved with 900 and 450 mg L^−1^ FeNPs at 24 and 48 h of exposure time, respectively. This is the first report of the effect of FeNP elicitor on hairy root cultures of a medicinal plant. We suggest that FeNPs could be an effective elicitor in hairy root cultures in order to increase tropane alkaloid production.

**Electronic supplementary material:**

The online version of this article (doi:10.1007/s11627-017-9802-0) contains supplementary material, which is available to authorized users.

## Introduction

**Figure 1.**
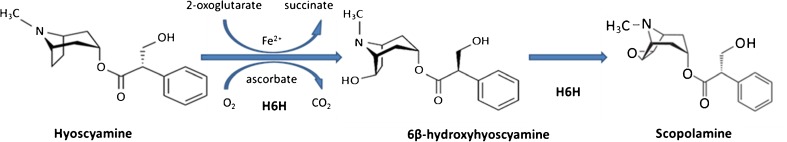
The last part of tropane alkaloid biosynthetic pathway.

*Hyoscyamus reticulatus* L. (belonging to Solanaceae family) is one of the most important medicinal plants in South-west Asia, Egypt, Iran, and Turkey (Madani *et al.* 2015). *Hyoscyamus* species are the main source of tropane alkaloids, especially scopolamine and hyoscyamine, which are commonly exploited in folk medicine. Due to the complicated chemical formulation of hyoscyamine and scopolamine, their synthetic production is too expensive and so, in practice, they are obtained from Solanaceae plants. They are normally produced in fresh root cells and transported to the aerial plant fragments (Ghorbanpour *et al.* 2015). *Agrobacterium rhizogenes*-induced genetically transformed root cultures in many Solanaceous species have revealed their potential for fast production of biomass with high contents of tropane alkaloids (Jouhikainen *et al.* 1999). For increased secondary metabolite production from medicinal plants, many approaches have been explored (Sharafi *et al.* 2013a, b; Mirzaee *et al.* 2016), such as selection of high yielding cell lines, growth media adaptation, elicitation, precursor feeding, large scale culture in bioreactor systems, hairy root culture, plant cell immobilization, and biotransformation. The generation of noteworthy pharmaceutical secondary metabolites in plant cultures based on modern techniques such as tissue culture or genetic transformation methods is an alternate method compared to the extraction from roots. Also, genetic engineering has become an interesting approach for manipulating and revealing regulatory aspects of alkaloid biosynthesis. Development of efficient protocols for induction of hairy roots from some medicinal plants was established in our laboratory by different strains of *A. rhizogenes* (Sharafi *et al.* 2013 a; Sharafi *et al.* 2014 a, b; Valimehr *et al.* 2014). Elicitation is an effective method for improving the low yields of medicinal plants’ secondary metabolite production. Elicitors are chemicals or biological factors which can induce physiological and morphological reactions and secondary metabolite enhancement. The uses of biotic and abiotic elicitors in hairy root cultures are the most suitable approach for increasing the productivity. Prior studies have described enhancement of secondary metabolite production by different elicitors in hairy root cultures of medicinal plants. Artemisinin production was increased from 1.67 mg to 2.86 mg g^−1^ dry wt in hairy root cultures of *Artemisia annua* using 900 mg L^−1^ Ag-SiO_2_ core-shell nanoparticles (Zhang *et al.* 2013). In another study on *Anisodus luridus* hairy root cultures, the scopolamine efflux reached to 6.2 times comparing to the non-elicitated roots achieved by adding acetylsalicylic acid (ASA) as a chemical elicitor (Qin *et al.* 2014). Scopolamine is synthesized from hyoscyamine by moderation of 6β-hydroxyhyoscyamine (Fig. 1) (Zhang *et al.* 2013). Tropane alkaloid production has been elicited in hairy root culture of solanaceous plants such as *Brugmansia candida* (Pitta-Alvarez *et al.* 2000), *Anisodus acutangulus* (Kai *et al.* 2012), and *Hyoscyamus niger* (Jaremicz *et al.* 2013). CaCl_2_ and hemicellulase can increase the intracellular hyoscyamine and scopolamine accumulation, release, and production in *B. candida* hairy roots (Pitta-Alvarez *et al.* 2000). Elicitation of suspension-cultured *Corylus avellana* L. cells by 5 ppm silver nanoparticles led to enhanced taxol production (Jamshidi *et al.* 2014). The atropine yield in hairy root cultures of *Datura metel* induced with nanosilver was increased to 1.147-, 1.117-, and 2.42-fold in comparison to the control samples after 12, 24, and 48 h of treatment, respectively (Shakeran *et al.* 2015). In *Hypericum perfuratum* cell suspension cultures, production of hypericin and hyperforin was induced significantly by zinc and iron nanooxides (Sharafi *et al.* 2013a, b). The highest content of glycyrrhizin was observed in *Glycyrrhiza glabra* seedlings after elicitation by CuO and ZnO nanoparticles (Oloumi *et al.* 2015).

Reactive oxygen species are produced by different physicochemical and biochemical reactions. Elicitation with different elicitors may lead to oxidative stress induction. Generally, plants are protected against oxidative stresses by means of a wide range of radical scavenging systems such as antioxidative enzymes like peroxidase (POD), superoxide dismutase (SOD), ascorbate peroxidase (APX), and catalase (CAT), as well as non-enzymatic compounds (Hatami and Ghorbanpour 2014). Oxidative tension is a general response related to all stresses leading to various secondary responses, such as secondary metabolite generation. Iron oxide nanoparticles diameters are between about 1 and 100 nm. Iron oxide nanoparticles are magnetite, either Fe_3_O_4_ or γ-Fe_2_O_3_. Because of their paramagnetic attributes and their possible usage in many fields, they have attracted research interest (Sharafi *et al.* 2013a, b).

Nanomaterials can promote some metabolism and reveal physiological answers but the underlying mechanisms are unknown (Hatami and Ghorbanpour 2014). To the best of our knowledge, no previous study has surveyed the influence of iron oxide nanoparticles as abiotic elicitor on enhancement of hyocyamine and scopolamine productivity in hairy root culture of *H. reticulatus* L. The main goal of this study is the evaluation of the antioxidant activity, growth, and production of hyoscyamine and scopolamine by elicitation with iron oxide nanoparticles at different concentrations and exposure times in hairy root culture of *H. reticulatus* L.

## Materials and Methods

### **Plant materials**

Seeds of *H. reticulatus* were provided by Pakan Bazr Company, Isfahan, Iran. *H. reticulatus* seeds were surface sterilized in 70% (*v*/*v*) ethanol and 10% (*v*/*v*) NaOCl and then washed three times in sterile water. Afterward, seeds were cultured in MS medium supplemented with 3% (*w*/*v*) sucrose, 7.2 g L^−1^ agar (Duchefa, Haarlem, Netherlands), and 0.1 g L^−1^ myo-inositol (Duchefa, Netherlands). One week after germinating, cotyledons were isolated as explants.

### **Hairy root induction and culture**

The explants (cotyledons) were infected with *A. rhizogenes* strain A7 and incubated in the dark on hormone free MS medium supplemented with 3% (*w*/*v*) sucrose, 7.2 g L^−1^ agar, and 0.1 g L^−1^ myo-inositol and after 48 h transferred to the same medium supplemented with 200 mg L^−1^ cefotaxim. After 2 weeks, hairy roots were induced and observed. They were sub-cultured every 10 d and after three passages transferred to antibiotic free MS medium. The cultures were transferred to 250 mL Erlenmeyer flasks (shaken at 120 rpm at 25°C in darkness) containing 30 mL hormone-free liquid MS medium and sub-cultured every 2 weeks.

### **Polymerase chain reaction analysis**

Total DNA was extracted from transformed and non-transformed roots using DNA isolation kit (Fermentas Vilnius, Germany). PCR analysis with specific primers of *rol* B gene was performed. The primers designed to amplify *rol* B were 5’-tggatcccaaattgctattccacga-3′and 5’-ttaggcttctttcttcaggtttactgcagc-3′. The PCR reactions contained, in a final volume of 20 μL of 1 × PCR buffer, 3 mM MgCl_2_, 1 mM of each dNTP (Fermentas Co.), 0.4 μM of each specific primer, 1 U of *Taq* DNA polymerase (Fermentas Co.), and 20 ng genomic DNA or 10 ng pRi plasmid DNA used as positive control. The PCR conditions were 94°C (5 min), 30 cycles of three steps [94°C (1 min), 58°C (1 min), and 72°C (30 s)], and 72°C (10 min) for final extension. PCR products were revealed following electrophoresis on 1% agarose under UV trans-illuminator.

### **Elicitor preparation and elicitation**

Iron oxide (Fe_3_O_4_) nanoparticle solution was provided by Nanozaino Co., Tehran, Iran. To investigate the influence of iron oxide nanoparticles (FeNPs), different concentrations of this elicitor (0, 450, 900, 1800, and 3600 mg L^−1^) were added to MS culture media (pH = 5.8 before autoclaving) of 10-days-old hairy roots of *H. reticulatus*. Hairy root culture was induced with FeNPs for 24, 48, and 72 h and then transferred to elicitor-free MS culture medium fortified with 3% (*w*/*v*) sucrose, 7 g L^−1^ agar, and 100 mg L^−1^ myo-inositol for growth and production of tropane alkaloids. Hairy roots were harvested after a week, air-dried, and milled for extraction of alkaloid.

### **Alkaloid extraction**

Alkaloid extraction was performed as described in Kamada *et al.* (1986). Briefly, 500 mg powdered sample was diluted with 10 mL solvent containing CHCl_3_/MeOH/25% (*w*/*v*) NH_4_OH (15:5:1 *v*/*v*/*v*) per 100 mg dry sample and sonicated for 10 min, kept at room temperature (1 h), and then filtered. The residue was washed twice with 1 mL of CHCl_3_ and dried. Five milliliters of CHCl_3_ and 2 mL of 1 N H_2_SO_4_ were added to the residue and mixed. The H_2_SO_4_ phase was adjusted to pH 10 with 28% (*w*/*v*) NH_4_OH in an ice bath and extracted once with 2 mL and twice with 1 mL of CHCl_3_. The combined aqueous extracts were dried over anhydrous Na_2_SO_4_, and then, the residue was washed with 1 mL of CHCl_3_. After evaporation, the extract was dissolved in 1–2 mL MeOH and subjected to GC-MS analysis. GC analysis was performed on a Hewlett–Packard (HP, Palo Alto, CA) HP 7890A. GC-MS analysis was based on Gharari *et al.* (2016) method.

### **Enzyme assay**

Enzyme extraction was performed as in Kang and Saltveit (2002). Antioxidant enzyme activity including catalase (CAT) was performed according to Maehly and Chance (1959), ascorbate peroxidase (APX) activity was determined according to Chen and Asada (1989) with minor modification, and guaiacol peroxidase (GPX) activity was determined according to Upadhyaya *et al.* 1985.

### **Statistical analysis**

The experiment was performed as a factorial based on completely randomized design with three replicates. One-way analysis of variance (ANOVA) was done and means compared using Duncan’s multiple range test at the 99% certainty level (*P* ≤ 0.01) using SAS 9.1 software.

## Results and Discussion

### **Induction and establishment of hairy root cultures**

Seeds were germinated after 5 days (Fig. S1A). The cotyledon explants, from 1-wk-old seedlings of *H. reticulatus* (Fig. S1B) were isolated (Fig. S1C) and infected with *A. rhizogenes* strain A7 (Fig. S1D). After 2 weeks, hairy roots were induced and appeared (Figs. S2A, S2B). Normal and rapid grown hairy roots in solidified MS media (Figs. S2C, S2D) were selected to establish hairy root lines in liquid MS media. Line 8 (L8) with normal morphological structures and stable growth was selected for the next experiments (Fig. S2E). Hairy roots were harvested a week after treatment for alkaloid extraction (Fig. S2F).

### **PCR analysis for molecular confirmation of transformation**

**Figure 2.**
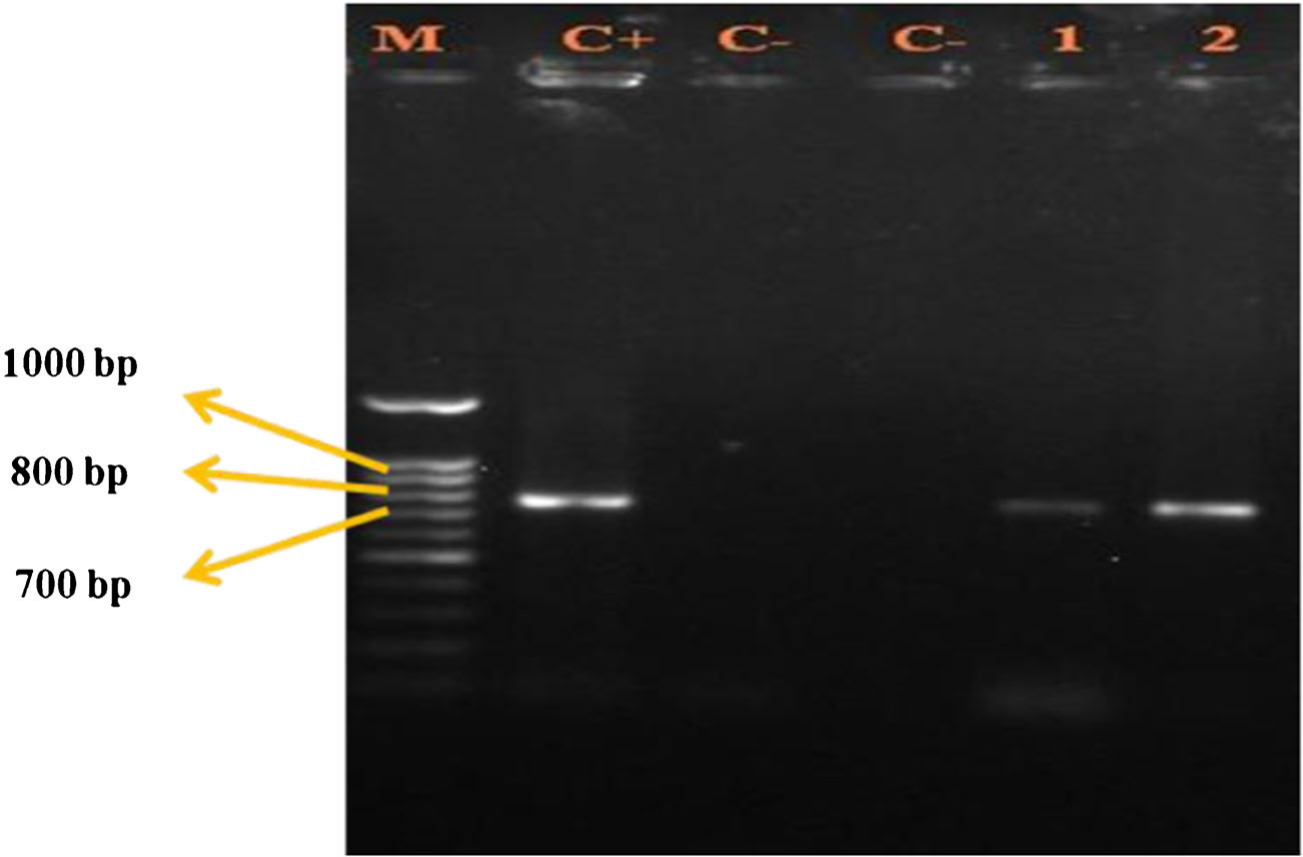
PCR analysis for detection of the *rolB* gene in normal and hairy root lines of *Hyoscyamus reticulatus L.*; *M* molecular size marker (1 kb ladder), *1* and *2* hairy root lines, (*C−*) negative control (non-transformed root and the PCR reaction without DNA template). (*C+*) positive control (Ri plasmid).

To probe the existence of the *rol* B gene conveyed from *A. rhizogenes* Ri plasmid, PCR analysis was conducted. Figure 2 shows PCR assay for identification of the *rol* B gene in two acquired hairy root lines of *H. reticulatus*. The PCR analysis of hairy roots produced an amplicon as well as the positive control, while no amplicon observed in the DNA extracted from *H. reticulatus* roots and negative control.

### **FeNP effects on hairy root growth and tropane alkaloid production**

**Figure 3.**
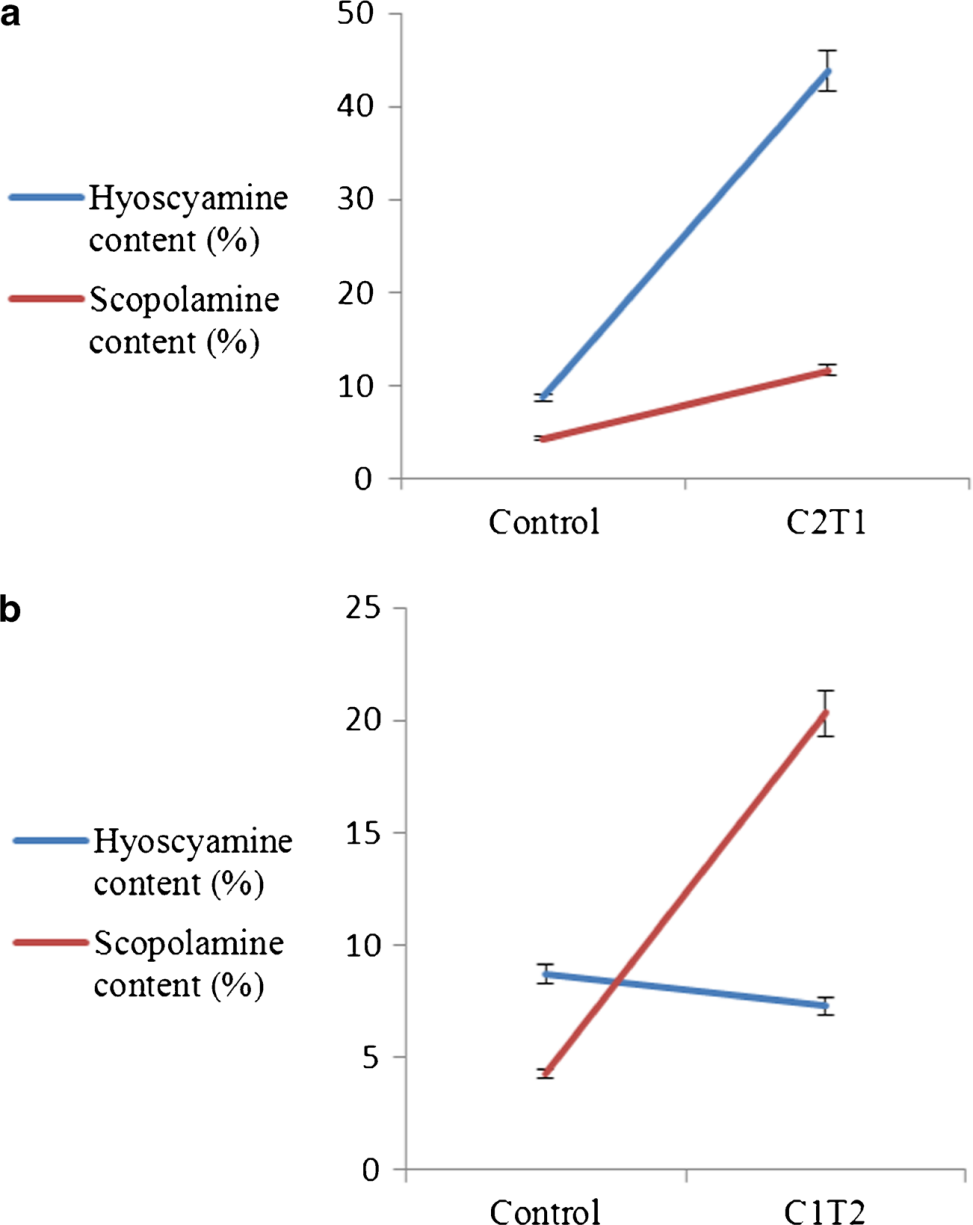
(*a*) Hairy root culture treated with 900 mg L^−1^ iron oxide nanoparticles for 24 h. (*b*) Hairy root culture treated with 450 mg L^−1^ iron oxide nanoparticles for 48 h. *Error bars* for standard errors (SE), *n* = 3.

ANOVA showed that the growth of *H. reticulatus* hairy roots had not been significantly affected by different exposure times and concentrations of FeNPs (Supplementary material, Table 1, *P* ≤ 0.01). The highest hairy root fresh and dry weights were found in the medium supplemented with 900 mg L^−1^ FeNPs (10.56 and 0.61 g, respectively). However, there were no significant differences among fresh and dry weights of treated hairy roots and control (9.25 and 0.52 g, respectively). Extracted materials were used for GC-MS analysis. (Fig. 3 and Figs. S3A, S3B).

**Figure 4.**
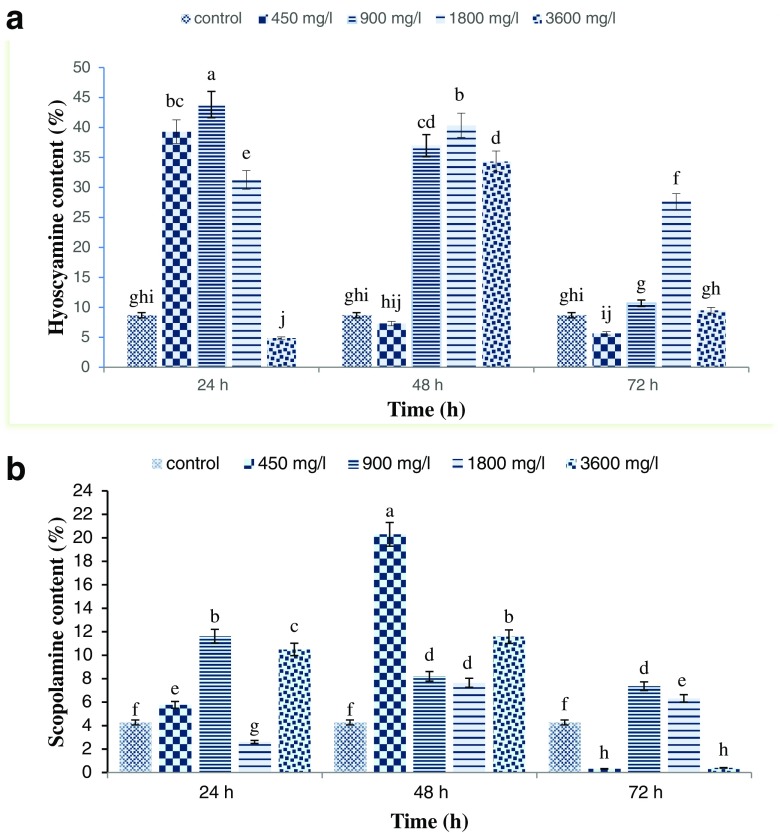
Effects of different concentrations of iron oxide nanoparticles at different exposure times on hyoscyamine (*a*) and scopolamine (*b*) content in hairy root culture of *Hyoscyamus reticulatus*. Mean values marked with *different letters* are significantly different according Duncan’s multiple range test (*P* ≤ 0.01). *Error bars* for standard errors (SE), *n* = 3.

GC-MS analysis revealed that elicitation with FeNPs at different concentrations and exposure times significantly affected content of hyoscyamine (Fig. 4 *a*) and scopolamine (Fig. 4 *b*) in hairy root cultures. The maximum hyoscyamine production was obtained in cultures subjected to 900 mg L^−1^ FeNPs for 24 h (43.82 vs. 8.69% in the control cultures, about fivefold increase). Elicitation with the highest FeNP concentration (3600 mg L^−1^) for 24 h resulted in minimum hyoscyamine production. The maximum scopolamine accumulation (20.3%) was observed in cultures elicited with 450 mg L^−1^ FeNPs for 48 h. The quantity of scopolamine in elicitated hairy roots with 450 and 3600 mg L^−1^ FeNPs for 72 h was decreased to 0.32 and 0.40%, respectively, compared to the scopolamine amount in the non-elicitated sample (4.27%).

The results showed that increasing exposure time significantly reduced hyoscyamine and scopolamine production. Increasing the treatment period decreased the secondary metabolite production, due to the toxic effects of nanoparticles on mitotic index (genotoxic) and DNA (Castiglione *et al.* 2011). Increasing the concentration of iron oxide nanoparticles resulted in a decline in tropane alkaloid production. The toxic effects of high concentrations of nanoparticles have been reported by several researchers (Yang and Watts 2005; Lin and Xing 2008; Sharafi *et al.* 2013 b).

The results demonstrated that hyoscyamine and scopolamine contents elicited in hairy roots with appropriate concentrations and exposure times were higher than the control. The results showed that iron oxide nanoparticles stimulated hyoscyamine and scopolamine production in *H. reticulatus* hairy root culture.

The last part of the tropane biosynthetic pathway is due to hyoscyamine-6-β-hydroxylase, which catalyzes the hydroxylation of hyoscyamine to scopolamine in two steps (Hashimoto and Yamada 1987). It seems that elicitation of *H. reticulatus* hairy root culture with iron oxide nanoparticles could make available sufficient Fe^2+^ required for this enzymatic reaction and increase the production of tropane alkaloids. Iron nanooxide is a novel elicitor of which there is no report available regarding utilization in hairy root culture of *H. reticulatus*. Nanoparticles on account of their physicochemical properties, e.g., enlarged surface area to volume, high surface reactivity, and ability to engineer electron exchange, can affect the redox status and modify the growth efficiency of plants (Mukherjee and Mahapatra 2009). For increasing tropane alkaloids, various techniques such as genetic engineering of key enzymes in biosynthetic pathway were analyzed. For example, engineered belladonna hairy roots with transgenic hyoscyamine-6β-hydroxylase gene recorded a fivefold-increased scopolamine production compared to native roots (Hashimoto *et al.* 1993). Over-expression of *pmt* and *h6h* gene in *Atropa belladonna* L. caused a huge increase (11 and 24 times) in hyoscyamine content in elicitated hairy roots compared to control and native roots, respectively (Yang *et al.* 2011).

The scopolamine levels in root cultures of *H. niger* after addition of 0.5 and 1 g L^−1^ yeast extract were increased (Hong *et al.* 2012). In *D. metel* hairy root culture, atropine content increased 2.4-fold after 48 h elicitation by nanosilver (Shakeran *et al.* 2015).

Activating specific genes and synthesis of alkaloids depends on various signaling molecules which interact with their related receptors in the plant plasma membrane. Biological or non-biological agents, used as elicitors, are responsible for triggering defense-related compounds through activation of specific transcription factors involved in secondary metabolite production. Jasmonate (JA) is one of the most important growth regulators which stimulate diverse plant defense responses, including the biosynthesis of secondary metabolites. It seems that nanoparticles may act in signal transduction paths that promote jasmonate production genes in cells under treatment (Sharafi *et al.* 2013 a).

Biochemical and GC-MS results revealed that elicitation by iron oxide nanoparticles had significant effects on the activity of key enzymes of tropane alkaloid biosynthesis such as putrescine *N*-methyltransferase (PMT) and hyoscyamine 6-β-hydroxylase (H6H). Also, elicitation directly or indirectly increased the *pmt* and the *h6h* gene expression leading to stimulation of tropane alkaloid production in hairy root cultures.

This study is the first report of FeNP application in hairy root culture of medicinal plants. Many of available reports about the *in vitro* application of nanoparticles relate to silver and other nanoparticles. The results of Sharafi *et al.* (2013 b) indicated an effective role of FeNPs in hypericine and hyperforine enhancement in cell suspension culture of *H. perforatum* L. Publications show that silver nanoparticles have an effective role in promotion of artemisinin producing in *A. annua* (Zhang *et al.* 2013), atropine in *D. metel* (Shakeran *et al.* 2015), and taxol in hazel cell suspension culture (Jamshidi *et al.* 2014). Cobalt and zinc nanoparticles increased the expression of genes related to the artemisinin biosynthetic pathway and have been proposed as elicitors to increase artemisinin content. Treatment of *G. glabra* L. seedlings with CuO and ZnO nanoparticles increased glycyrrhizin contents (Oloumi *et al.* 2015). Also, nanosized titanium dioxide had positive effects on tropane alkaloid production in *H. niger* L. plants. The results of this current study confirmed the enhanced production of hyoscyamine and scopolamine in *H. reticulatus* Lhairy root culture, elicited by FeNPs, and are in accordance with the results of research detailed above.

### **Effect of FeNPs on antioxidant enzyme activity of*H. reticulatus*hairy root cultures**

**Figure 5.**
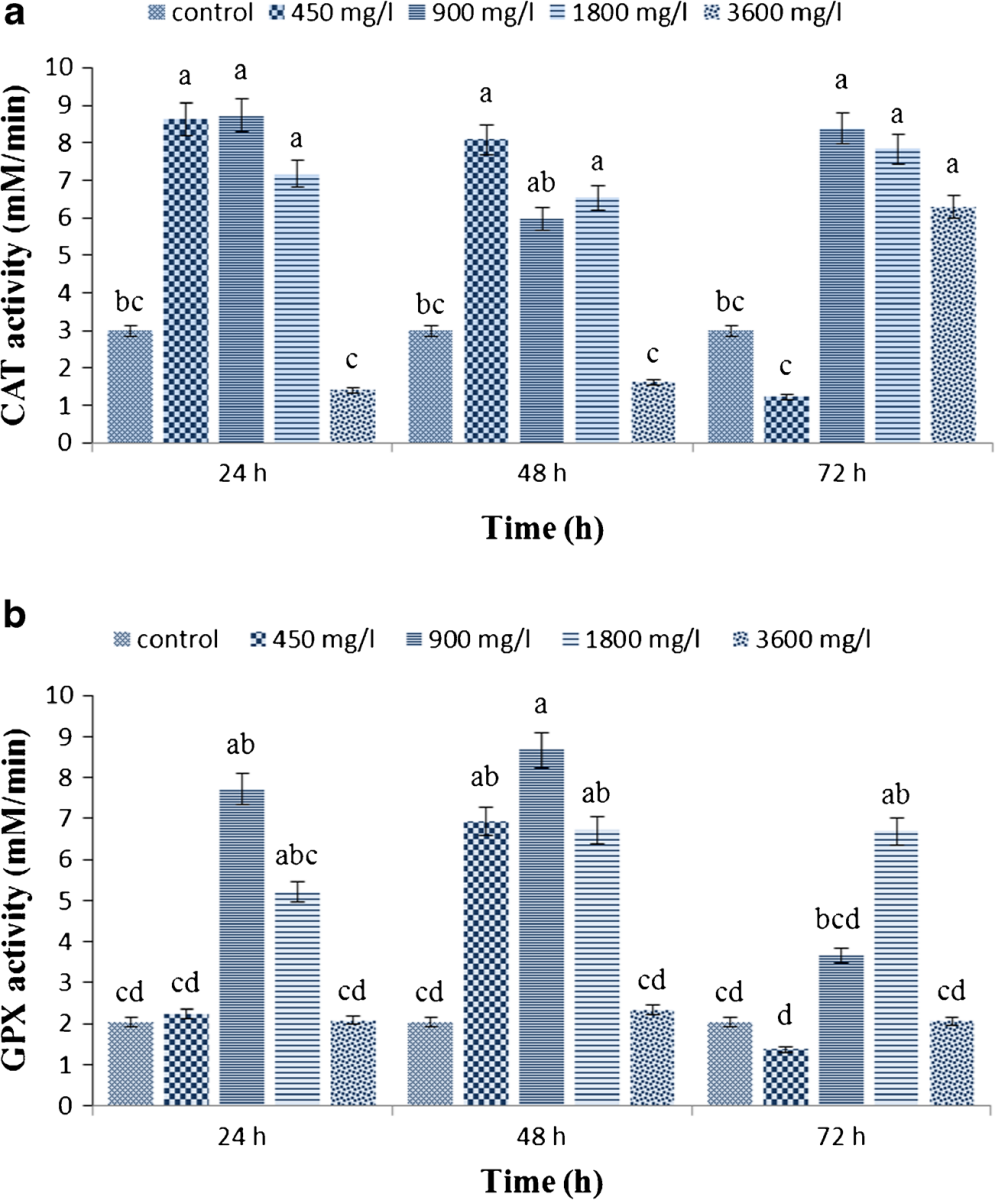
Effects of different concentrations of iron oxide nanoparticles at different exposure times on catalase (*a*) and guaiacol peroxidase (*b*) activity in hairy root culture of *Hyoscyamus reticulatus.* Mean values marked with *different letters* are significantly different according Duncan’s multiple range test (*P* ≤ 0.01). *Error bars* for standard errors (SE), *n* = 3.

Antioxidant enzyme activity was significantly increased in induced hairy roots compared to non-transgenic roots (Supplementary material, Table 2). The results revealed that elicitation of hairy root cultures with FeNPs at different concentrations and exposure times significantly (*P* ≤ 0.01) affected CAT and GPX activity, while there was no notable difference in the function of APX. Significant variations in antioxidant enzymes activity between the elicited hairy roots were detected. The highest CAT and GPX activity was detected in hairy root cultures exposed to 900 mg L^−1^ FeNPs for 24 and 48 h, respectively, and the lowest activity of both enzymes obtained after elicitation with 450 mg L^−1^ FeNPs for 72 h (Fig. 5 *a*, *b*).

Ascorbate is a substrate of APX in the final steps of the tropane alkaloid biosynthetic pathway (Fig. 1). As a result, APX activity was not significantly affected by elicitation. Elicitation by iron oxide nanoparticles lead to induction of oxidative stress. Most secondary metabolites from medicinal plants are defensive metabolites and can be stimulated by various elicitors. Hence, production of ROS by FeNPs as elicitor can lead to increased production of tropane alkaloids.

## Conclusion

The results of this study proved that use of iron oxide nanoparticles as abiotic elicitor was an effective method for enhancement of tropane alkaloids. According to the results, exposure of hairy root cultures of *H. reticulatus* to 900 mg L^−1^ FeNPs for 24 h and 450 mg L^−1^ FeNPs for 48 h was the best treatments for enhancement of hyoscyamine and scopolamine, respectively. This study is the first report of the application of FeNPs in hairy root culture. Results of these and other studies on nanosized particles demonstrated enhancement of secondary metabolite production. It seems that use of nanoparticles as abiotic elicitors could be an effective strategy to increase productivity of pharmaceutical compounds in medicinal plants.

## Electronic supplementary material

ESM 1 (DOC 3381 kb)
