# Supplementary material for: Enhanced production of hyoscyamine and scopolamine from genetically transformed root culture of Hyoscyamus reticulatus L. elicited by iron oxide nanoparticles
Source: In Vitro Cell Dev Biol Plant. 2017 Feb 27;53(2):104–11. doi: 10.1007/s11627-017-9802-0 (PMC5423962; doi:10.1007/s11627-017-9802-0)
Supplement: Supplementary file 1 — (DOC 3381 kb) [file 11627_2017_9802_MOESM_ESM.doc]

**Supplementary Material**

**Table 1**. Influence of elicitor and exposure time on tropane alkaloids content in *Hyoscyamus reticulatus* hairy roots.

| Mean of Squares | | Degree of Freedom | Variation Sources |
| --- | --- | --- | --- |
| Scopolamine content | Hyoscyamine content |  |  |
| 38.98^**^ | 951.89 ^**^ | 4 | Elicitor concentration (a) |
| 166.69^**^ | 862.16 ^**^ | 2 | Exposure time (b) |
| 75.74^**^ | 505.04^**^ | 8 | (a×b) |
| 20 | 1.54 | 30 | Standard Error |
| 6.44 | 5.86 |  | Coefficient of Variation |

**Table 2:** Influence of different elicitors on *Hyoscyamus reticulatus* hairy root fresh and dry weight and antioxidant activity.

|  | Mean of Squares | | |  | | Degree of Freedom | | Variation Sources |
| --- | --- | --- | --- | --- | --- | --- | --- | --- |
| Total antioxidant activity | | Dry Weight | Fresh Weight | |  | |  | |
| 630.59^**^ | | 0.0134^ns^ | 3.23 ^ns^ | | 4 | | Elicitor concentration (a) | |
| 1110.86^**^ | | 0.0023^ns^ | 1.14^ns^ | | 2 | | Exposure time (b) | |
| 213.57^**^ | | 0.0043^ns^ | 2.05^ns^ | | 8 | | (a×b) | |
| 6.21 | | 0.0055 | 3.23 | | 30 | | Standard Error | |
| 10.04 | | 13.38 | 18.67 | |  | | Coefficient of Variation | |


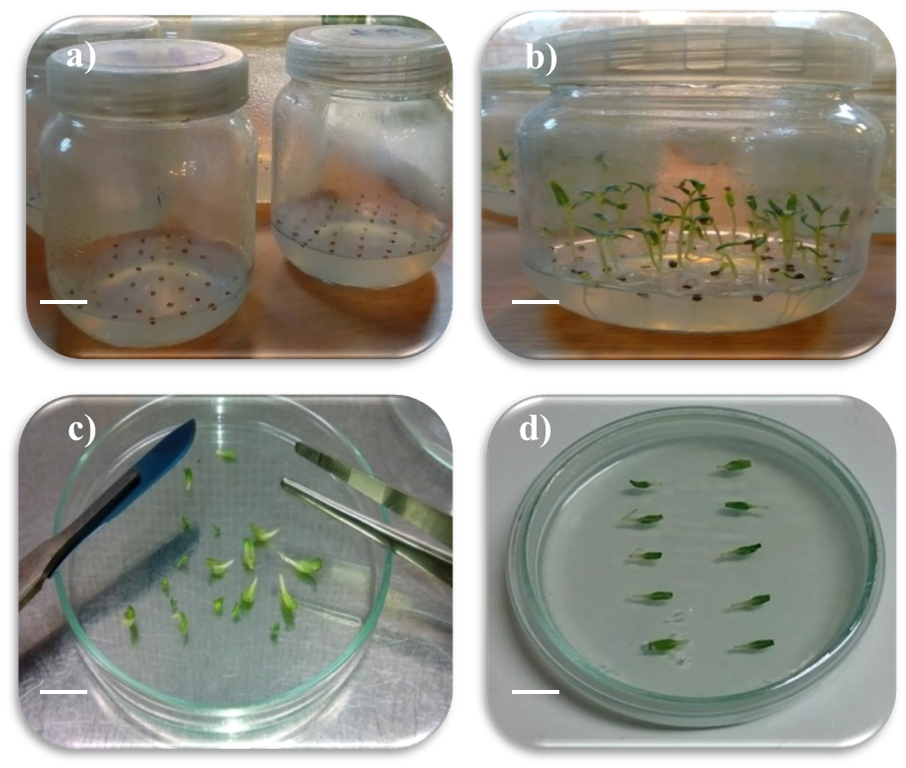


**Supplementary Figure 1.** Hairy root induction from *Hyoscyamus reticulatus* cotyledon explants; ***a*)** Seed culture on MS medium; ***b*)** 1-week-old Seedlings; ***c*)** cotyledon explants isolation; ***d*)** infected explants with *Agrobacterium rhizogenes* (bars= 2 cm)

| 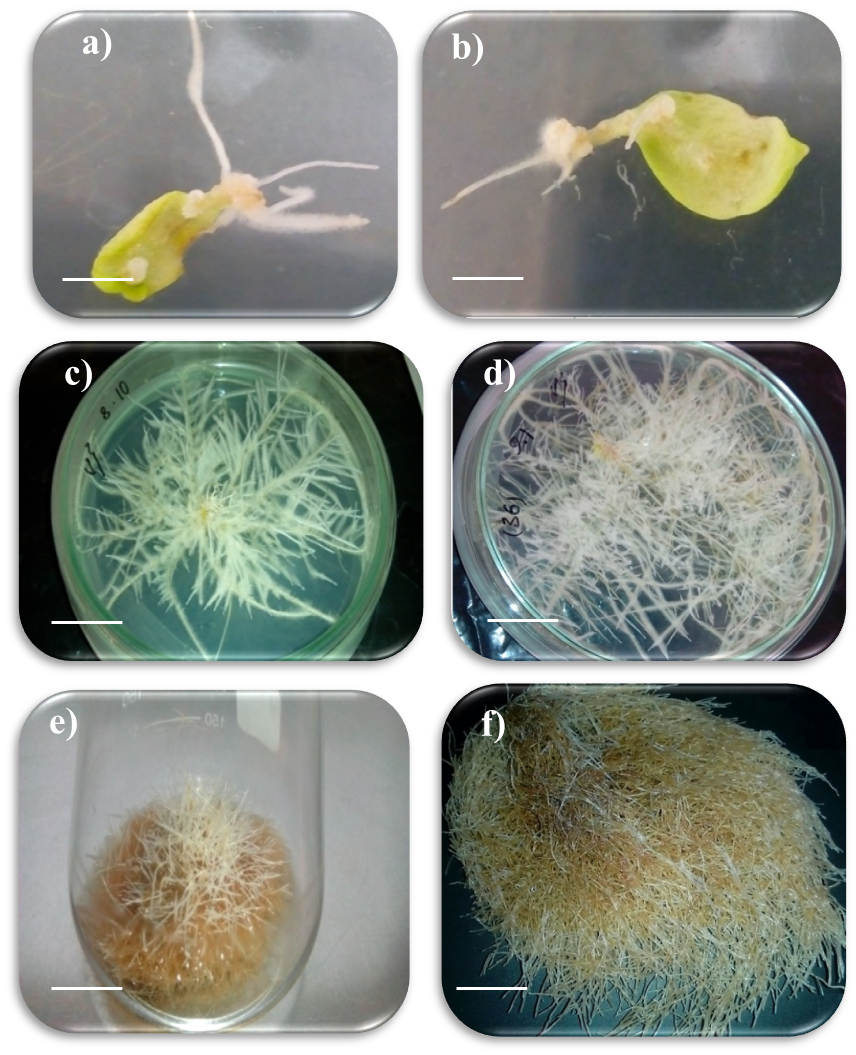 |
| --- |
| **Supplementary Figure 2.** *Agrobacterium rhizogenes* mediated transformation in *Hyoscyamus reticulatus* L.; **A, B)** hairy root induction on cotyledon explant after 2 weeks using *A. rhizogenes* strain A7; **C, D)** hairy root lines grown on MS medium; **E)** hairy roots transferred to 250 ml Erlenmeyer flasks; **F)** harvested hairy roots from liquid MS medium (*bars*= 5 mm) |


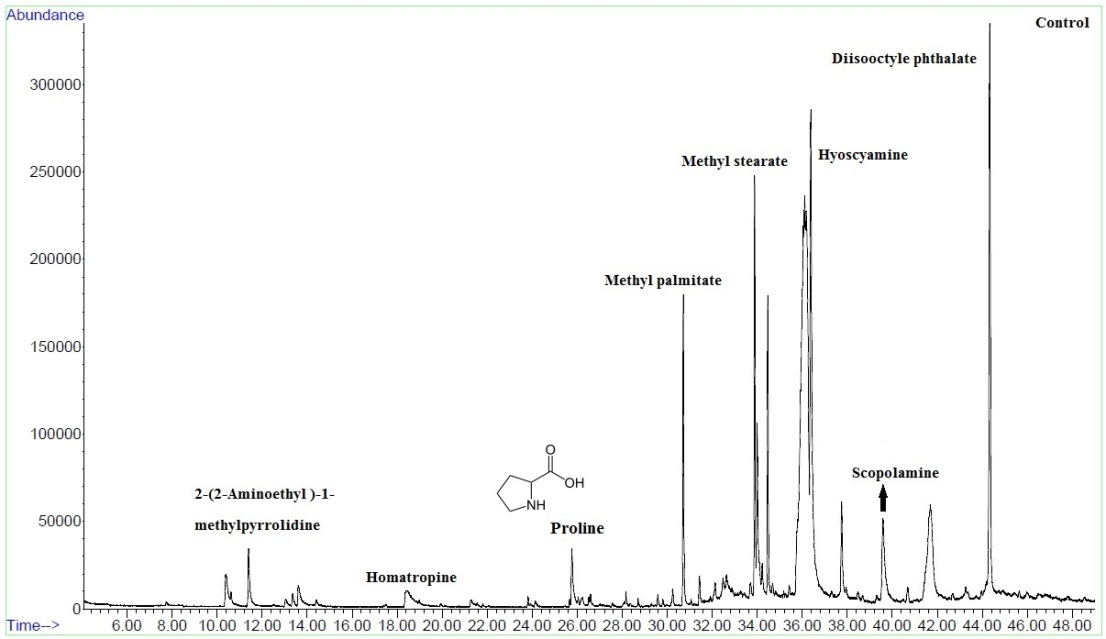


**a)**


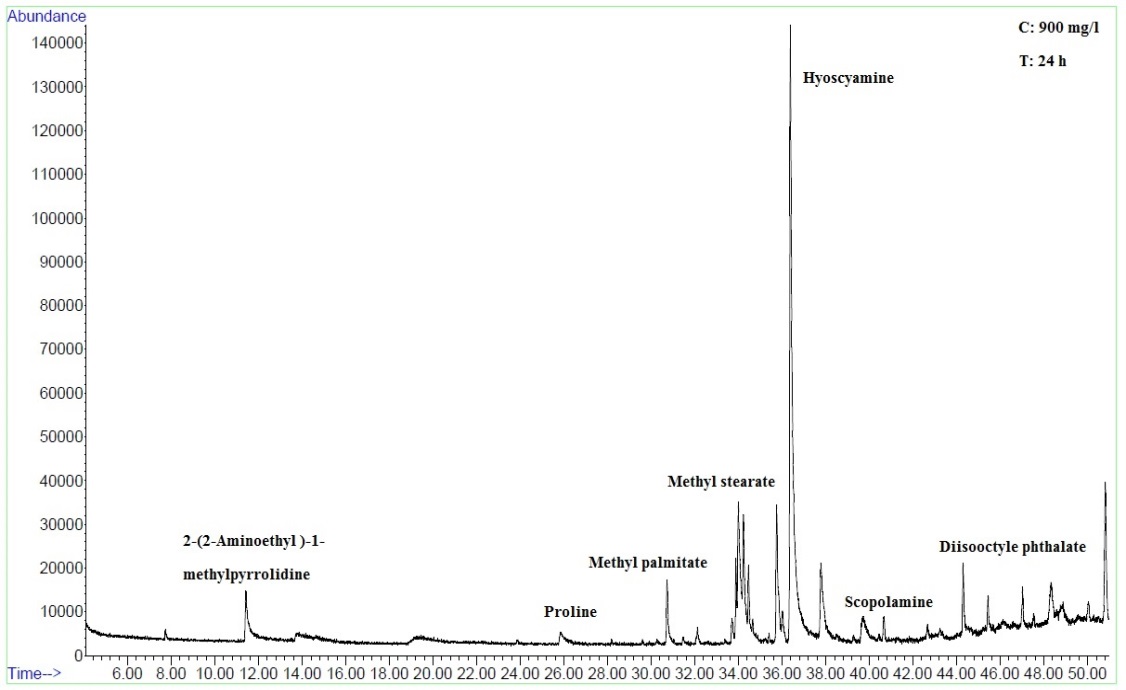


**b)**


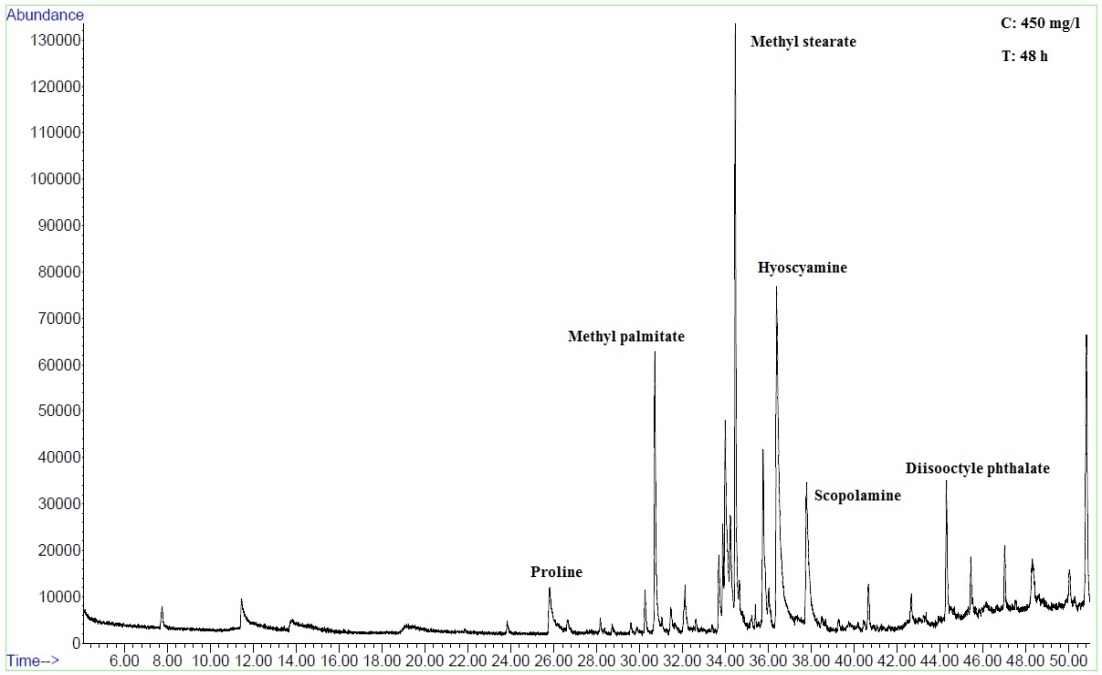


**c)**

**Supplementary Figure 3.** GC-MS analysis of tropane alkaloids: **A)** non-treated hairy roots; **B)** hairy roots culture treated with 900 mg L^-1^ iron oxide nanoparticles for 24 h; **C)** hairy roots culture treated with 450 mg L^-1^ iron oxide nanoparticles for 48 h
